# Gynaecological Causes of Acute Pelvic Pain: Common and Not-So-Common Imaging Findings

**DOI:** 10.3390/life13102025

**Published:** 2023-10-09

**Authors:** Paolo Niccolò Franco, Alejandra García-Baizán, María Aymerich, Cesare Maino, Sofia Frade-Santos, Davide Ippolito, Milagros Otero-García

**Affiliations:** 1 Department of Radiology, Hospital Universitario de Vigo, Carretera Clara Campoamor 341, 36312 Vigo, Spain; alejandra.garcia.baizan@sergas.es (A.G.-B.); sofiafradedossantos@gmail.com (S.F.-S.); maria.milagros.otero.garcia@sergas.es (M.O.-G.); 2 Department of Diagnostic Radiology, IRCCS San Gerardo dei Tintori, Via Pergolesi 33, 20900 Monza, Italy; mainocesare@gmail.com (C.M.); davide.atena@tiscalinet.it (D.I.); 3 Diagnostic Imaging Research Group, Radiology Department, Galicia Sur Health Research Institute (IIS Galicia Sur), Galician Health Service (SERGAS)-University of Vigo (UVIGO), 36213 Vigo, Spain; maria.aymerich@iisgaliciasur.es; 4 Instituto Português de Oncologia de Lisboa Francisco Gentil (IPOLFG), Rua Prof. Lima Basto, 1099-023 Lisbon, Portugal; 5 School of Medicine, University of Milano Bicocca, Via Cadore 33, 20090 Monza, Italy

**Keywords:** diagnostic radiology, ultrasounds, computed tomography, magnetic resonance imaging, acute pelvic pain, gynaecology

## Abstract

In female patients, acute pelvic pain can be caused by gynaecological, gastrointestinal, and urinary tract pathologies. Due to the variety of diagnostic possibilities, the correct assessment of these patients may be challenging. The most frequent gynaecological causes of acute pelvic pain in non-pregnant women are pelvic inflammatory disease, ruptured ovarian cysts, ovarian torsion, and degeneration or torsion of uterine leiomyomas. On the other hand, spontaneous abortion, ectopic pregnancy, and placental disorders are the most frequent gynaecological entities to cause acute pelvic pain in pregnant patients. Ultrasound (US) is usually the first-line diagnostic technique because of its sensitivity across most common aetiologies and its lack of radiation exposure. Computed tomography (CT) may be performed if ultrasound findings are equivocal or if a gynaecologic disease is not initially suspected. Magnetic resonance imaging (MRI) is an extremely useful second-line technique for further characterisation after US or CT. This pictorial review aims to review the spectrum of gynaecological entities that may manifest as acute pelvic pain in the emergency department and to describe the imaging findings of these gynaecological conditions obtained with different imaging techniques.

## 1. Introduction

Acute pelvic pain (APP) is defined as lower abdominal or pelvic pain of less than three months’ duration [1]. In female patients, acute pelvic pain may be caused by gynaecological, gastrointestinal, and urinary tract pathologies. Due to the variety of diagnostic possibilities, the correct assessment of these patients may be challenging. The most frequent gynaecological causes of acute pelvic pain in non-pregnant women are pelvic inflammatory disease, ruptured ovarian cysts, ovarian torsion, and degeneration or torsion of uterine leiomyomas [2,3,4,5]. On the other hand, spontaneous abortion, ectopic pregnancy, and placental disorders are the most frequent gynaecological entities that cause acute pelvic pain in pregnant patients (Table 1) [4,6,7]. Non-gynaecological causes, such as acute appendicitis, cystitis, diverticulitis, and nephrolithiasis, should always be considered [8]. 

Another common clinical manifestation of gynaecological and obstetrical pathologies that is often associated with APP is vaginal bleeding. It can have a variety of causes, ranging from benign conditions to severe medical disorders. From a clinical point of view, vaginal bleeding can be divided into menstrual, postmenopausal, and intermenstrual bleeding [9]. Each of them can be caused by different pathological conditions, including hormonal imbalance, infection and inflammation, miscarriage, ectopic pregnancy, and gynaecological malignancies [10,11,12,13,14]. 

In the case of patients presenting with APP, the diagnosis is based on anamnesis, clinical and laboratory findings, and diagnostic imaging. Determining patients’ pregnancy status is essential to aid the diagnosis. Ultrasound (US) is usually the first-line diagnostic technique because of its sensibility across most common aetiologies and its lack of radiation exposure [15,16]. Computed tomography (CT) may be performed if ultrasound findings are equivocal, if a gynaecologic disease is not initially suspected, or if it extends beyond the field of view achievable with US examination [15,17]. Magnetic resonance imaging (MRI) is an extremely useful second-line technique for the further characterisation after US or CT [18,19]. 

On these bases, this pictorial review aims (1) to summarise the spectrum of gynaecological entities that may manifest as acute pelvic pain both in pregnant and non-pregnant women; (2) to identify and describe the most characteristic imaging findings of these gynaecological conditions obtained with different imaging techniques; (3) to outline the most appropriate management of patients with acute pelvic pain underlying the differences between pregnant and non-pregnant patients; and (4) to propose a flowchart of acute pelvic pain imaging management in the emergency department (ED).

## 2. Differential Diagnosis

### 2.1. Ovarian Torsion

Ovarian torsion (OT), also known as adnexal torsion or tubo-ovarian torsion, is an emergency condition that occurs when the ovary twists around its vascular pedicle, frequently with a portion of fallopian tubes [20]. It is considered a relatively frequent condition, accounting for 3% of all gynaecological surgical emergencies. It occurs in females of all ages but is most common in women of childbearing age. Torsion is often associated with the presence of cysts or benign ovarian tumours, with a size greater than 5 cm being considered a risk factor. However, due to ovarian hypermobility, it can occur with no underlying ovarian pathology. There is also an increased risk of OT during pregnancy, most frequently between the 6th and 14th week of pregnancy. OT typically manifests as acute and severe unilateral lower abdominal pain that is accompanied by other symptoms, such as nausea, vomiting, and fever [20,21]. If torsion is suspected, immediate diagnosis is essential to avoid infarction and preserve ovarian function [22,23].

**Figure 1:**
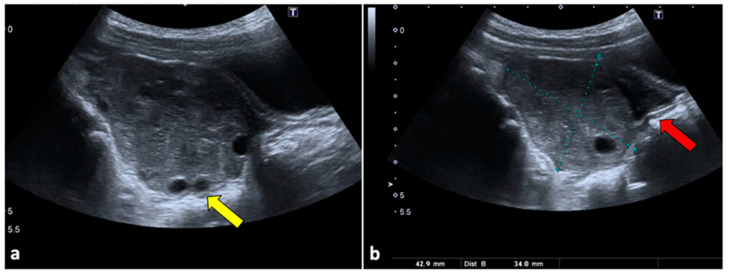
Ovarian torsion in an eight-year-old patient presenting at the emergency department with aching right pelvic pain for a few hours. Transabdominal US examination (**a**,**b**) shows that the right ovary is grossly enlarged (long axis: 42.9 mm; short axis 34 mm) and abnormally located in the midline. The ovary appears slightly echogenic, with many small cysts at the periphery (yellow arrow). Small volume free fluid with tiny echoes is present in the pelvis (red arrow). During the Doppler US evaluation, no vascular flow was noticed (not shown).

US combined with colour Doppler is the first-line imaging of choice, and the findings depend on the duration and severity of the torsion. Typical findings are an enlarged (>4 cm), oedematous, and heterogeneous ovary found in an unusual location, for instance, shifted medially or cranially to the uterine fundus. The ovary usually shows a hyperechoic central stroma with peripherally displaced follicles (follicular ring sign) [24]. An underlying ovarian lesion may be seen. Upon the Doppler evaluation, a reduced or absent vascular flow is typically observed, and the twisted vascular pedicle could be seen as whirlpool sign. Free fluid is often observed (Figure 1) [25,26]. 

**Figure 2:**
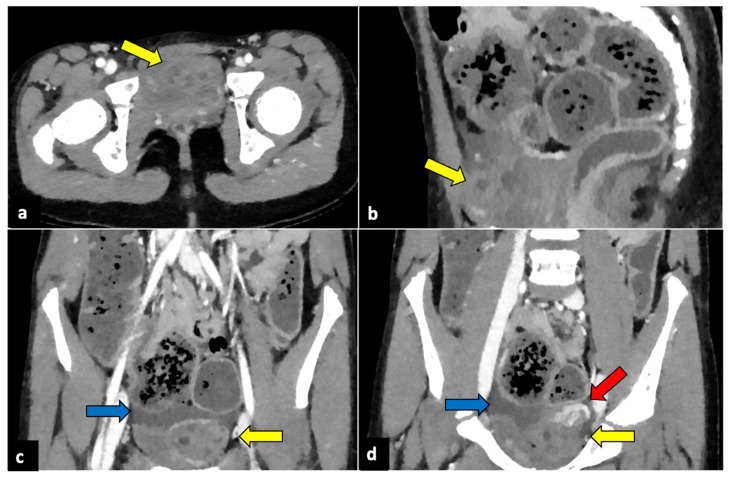
Left ovarian torsion in a 12-year-old woman with acute lower abdominal pain and vomiting. Axial (**a**), sagittal (**b**), and coronal (**c**,**d**) contrast-enhanced CT images show enlarged and oedematous left adnexa migrated to the midline and anteriorly to the uterus (yellow arrows), with peripherally displaced ovarian follicles. A small amount of free pelvic fluid (blue arrows) is associated. On the coronal plane, the twisted and oedematous ovarian vascular pedicle (red arrow) can be easily detected.

Although CT is not indicated as a first-line technique when OT is suspected, it is usually performed in female patients with aching nonspecific pelvic pain and vomiting. Moreover, it can be helpful for ruling out OT if US does not show ovarian abnormalities or if it is limited due to patient pain. CT scans usually show a large pelvic mass abnormally located at the midline, anteriorly to the uterus. During no-contrast scans, the ovary may appear hyperdense (>50 Hounsfield Unit) due to internal haemorrhage. In contrast-enhanced images, the ovarian twisted pedicle may be detected [27,28]. It can also be seen as a triangular enhancing soft tissue between the uterus and the involved ovary. Peripherally displaced follicles, fat stranding near the adnexa, and a small amount of free fluid are also common findings (Figure 2) [26].

**Figure 3:**
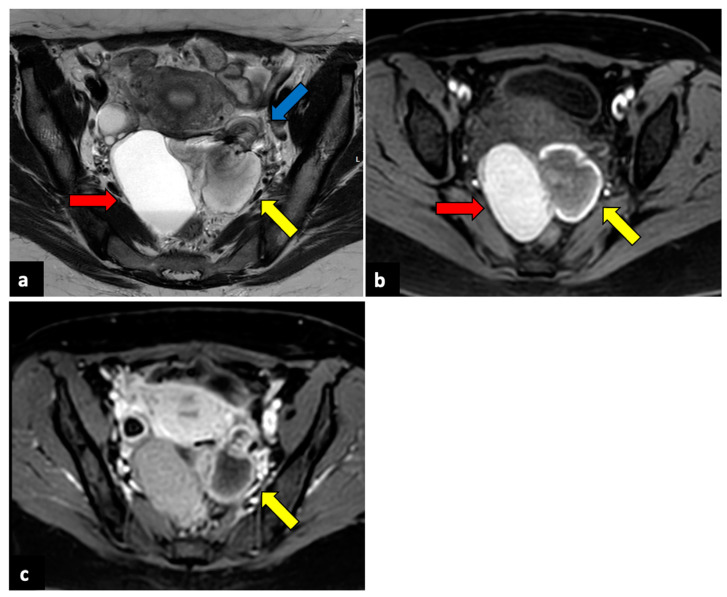
Left ovarian torsion in a thirty-nine-year-old patient with acute pelvic pain. Axial T2-weighted (**a**) and T1-weighted fat saturated before (**b**) and after (**c**) gadolinium MR pelvic images show an enlarged left ovary located in the Douglas pouch (yellow arrows), with and adjacent twisted vascular pedicle (“whirlpool sign”, blue arrow). The ovary presents a peripheral high signal on T1 extending to the pedicle (rim of methaemoglobin) without enhancement upon post-contrast sequences. A large cystic lesion arises from the left ovary with an internal high signal on both T1 and T2 (red arrows) and declivous sediment of low signal on T2, suggestive of a haemorrhagic cyst. The patient underwent a laparoscopic left adnexectomy, and pathology confirmed the diagnosis of ovarian torsion possibly due to the haemorrhagic cyst.

MRI is usually not available in emergency settings. However, it can be useful for further evaluating equivocal findings seen with other techniques and for characterising an underlying ovarian mass [26]. If haemorrhagic infarction is present, the involved ovary shows a T1 hyperintense signal during MRI, with low or without contrast enhancement, and can show diffusion restriction on Diffusion-Weighted Imaging (DWI) sequences. A T1 thin hyperintense ring, representing methaemoglobin, may also be present. Furthermore, the oedematous twisted pedicle can be seen as a heterogeneous structure with a high T2 signal located between the uterus and the enlarged ovary (Figure 3) [28,29].

### 2.2. Ovarian Cyst Haemorrhage/Rupture

**Figure 4:**
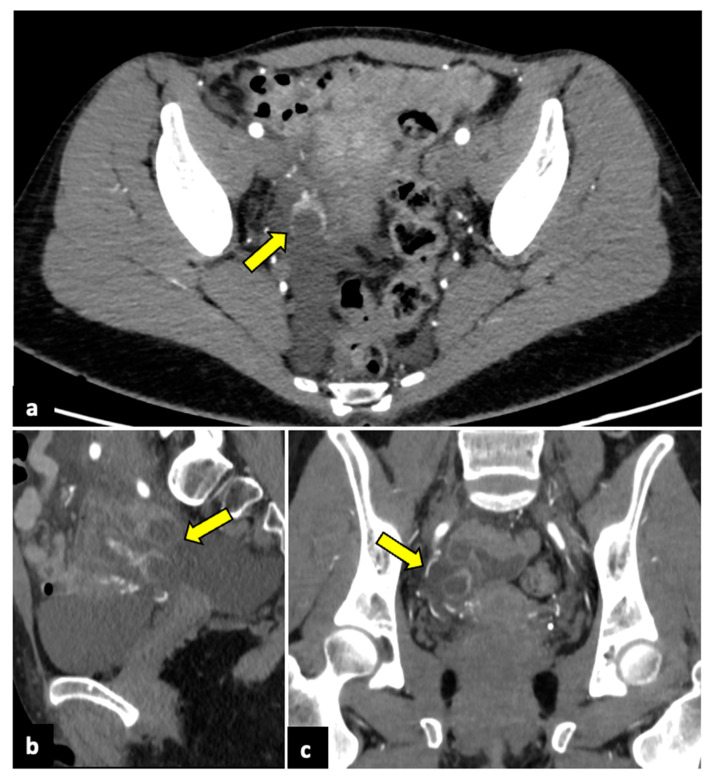
Ruptured ovarian corpus luteal cyst. CT axial (**a**), sagittal (**b**), and coronal (**c**) scans of a twenty-eight-year-old woman with acute right-sided pelvic pain. HCG test was negative. Images show a hypodense cystic lesion in the right adnexa, with thick and enhancing walls (yellow arrows). A focal discontinuity in the posterior wall is observed. Free pelvic fluid is also visible. The patient was treated conservatively.

Acute bleeding or rupture of functional (follicles and corpus luteum) ovarian cysts is one of the most common causes of APP in young women [30]. In severe cases, it can be associated with haemorrhagic shock and hypotension and, thus, can be potentially life-threatening. Clinically, it may overlap with an ectopic pregnancy, which should be ruled out by evaluating serum human chorionic gonadotropin (hCG) levels. In the case of acute haemorrhage, upon a US evaluation, the cyst is isoechoic in comparison with the ovarian stroma. It can mimic an enlarged ovary, while a thick irregular wall of increased peripheral vascularity (“ring of fire”) may be seen during a colour Doppler US [31]. In case of cyst rupture, chronic bleeding, septations, and liquid-sediment levels can be detected. At the CT evaluation, haemorrhagic cysts are typically heterogenous lesions with hyperdense areas. If a haemorrhagic cyst ruptures, hyperdense ascites is generally seen in the pouch of Douglas and sometimes in the upper abdomen (Figure 4) [16,32]. During MRI, haemorrhagic cystic lesions typically show a high signal on T1-weighted images and an intermediate-to-low signal on T2-weighted ones. In the case of haemorrhagic cyst ruptures, haemoperitoneum can be seen as free peritoneal fluid characterised by areas of both low and high signal intensity depending on the extent of blood-clot formation [5].

### 2.3. Pelvic Inflammatory Disease

Pelvic inflammatory disease (PID) is one of the most common causes of APP and typically occurs in young and sexually active women [4,33]. PID includes many conditions, such as endometritis, salpingitis, pyosalpinx, tubo-ovarian abscess (TOA), and pelvic peritonitis [34]. Clinical symptoms’ spectrum is very wide, varying from asymptomatic infection to abdominal pain, malaise, fever, vaginal bleeding, dysuria, or dyspareunia. PID is usually caused by ascending sexually transmitted infections that may involve the endometrium, fallopian tubes, ovaries, and peritoneum. The most common involved pathogens are Chlamydia trachomatis or Neisseria gonorrhoeae [35]. PID pathogens have an important role in producing tubal damage and in the development of adverse sequelae such as infertility or ectopic pregnancy. Thus, early diagnosis and treatment are crucial for preserving fertility and avoiding long-term consequences such as chronic pelvic pain and an increased risk of ectopic pregnancy, since the effectiveness of antibiotic therapy is dependent upon the interval from the onset of symptoms to the initiation of treatment [36]. The diagnosis is based on clinical and laboratory parameters, but imaging plays a crucial role, especially in the case of atypical clinical presentations [36,37]. 

Normal fallopian tubes cannot usually be distinguished upon imaging. In the case of salpingitis, lumen occlusion leads to the retention of purulent secretion and tubes swelling (pyosalpinx). During a US, a tubular structure with hyperechoic content is usually seen in the adnexal area. CT and MRI allow for a better visualisation of the dilated tube. Pyosalpinx is observed as a contrast-enhancing parauterine tubular structure with a thickened wall and incomplete septa (“cogwheel sign”) [38,39,40,41].

**Figure 5:**
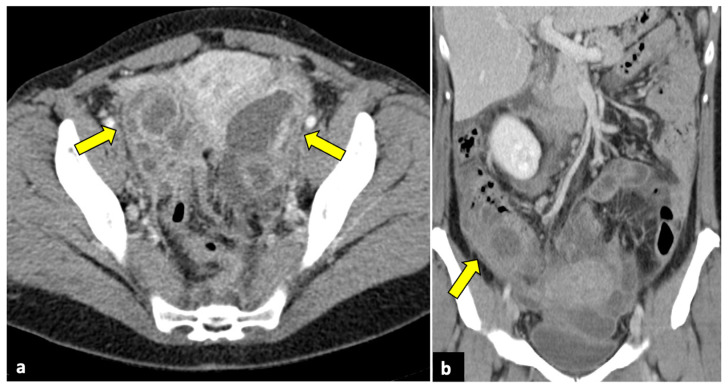
Bilateral tubo-ovarian abscesses in a 44-year-old woman who presented with fever, leucocytosis, and acute pelvic pain. CT axial (**a**) and coronal (**b**) pelvic scans demonstrate thick-walled, peripherally enhancing, multi-cystic, and tubular structures (yellow arrows), which proved to be bilateral tubo-ovarian abscesses at the time of surgery.

When the inflammatory process evolves from the fallopian tubes to the ovaries, it produces a condition known as tubo-ovarian abscess (TOA) [42]. Although US is considered the first method of choice for the diagnosis of PID, contrast-enhanced CT is often needed to confirm the diagnosis of an abscess, assess the extent of peritoneal disease, evaluate possible complications, plan treatment, and exclude non-gynaecological causes. Moreover, multiplanar sequences facilitate the visualisation of the tubular morphology of enlarged tubes. The typical imaging findings of a TOA are bilateral, centrally hypodense, multilocular, thick-walled adnexal lesions (Figure 5). Identifying a pyosalpinx as a tortuous, tubular lesion is essential for the diagnosis of TOA. Frequent findings include free fluid, pelvic peritonitis, and a fluid-filled uterine cavity [38,43]. 

On the contrary, MRI is rarely needed to diagnose PID and is more likely to be used for a better delineation of uterus and adnexal structures, the differential diagnosis of unclear adnexal lesions, and the differentiation of PID from other pathologic processes. During MRI, TOA usually presents with a hypointense signal on T1-weighted sequences and a hyperintense signal on T2-weighted sequences. A T1 hyperintense rim along the inner wall of the abscess cavity, because of the presence of haemorrhage or granulation tissue, is often detected. However, the MRI findings depend on the presence of blood and the protein content of the mass. MRI is also a useful tool for distinguishing pyosalpinx from haematoalpinx because the latter usually does not demonstrate wall thickening and has a higher signal intensity on T1-weighted images due to the presence of blood products [38]. 

In approximately 4% of cases during PID, right upper quadrant abdominal pain may occur due to inflammation of the hepatic capsule and overlying peritoneum with adhesion formation (perihepatitis). This condition is known as Fitz–Hugh–Curtis syndrome, and during CT imaging, it manifests as a thickened and enhanced liver capsule, transient hepatic perfusion abnormalities in arterial phases, periportal oedema, and inflammatory stranding in the right paracolic gutter [44,45].

### 2.4. Complicated Uterine Leiomyomas 

Uterine leiomyomas are extremely common benign and usually asymptomatic gynaecological tumours. However, they can undergo several complications and cause APP [46,47].

Red degeneration, also referred to as carneous degeneration, is one of four main types of degeneration that can involve uterine fibroids and is the most frequently occurring type during pregnancy, particularly during the second and third trimesters, and in the case of large fibroids (>5 cm) [48].

**Figure 6:**
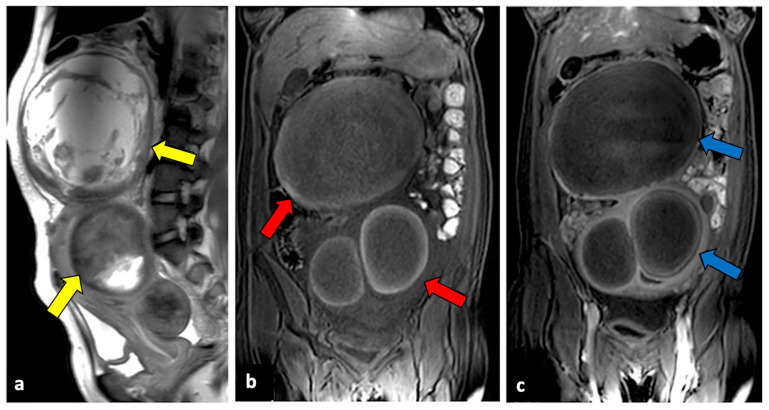
Red degenerated leiomyoma in a thirty-one-year-old woman with abdominal pain and vaginal bleeding after a miscarriage. T2-weighted imaging (**a**) showed an enlarged uterus with degenerated intramural and pedunculated leiomyomas with inhomogeneous intensity (yellow arrow). On T1-weighted sequence (**b**), a peripheral hyperintense rim surrounding the lesion’s central area of lower signal intensity (red arrows), typical of red degeneration of leiomyomas, is observed. No intralesional enhancement was seen after intravenous contrast injection ((**c**), blue arrows).

Red degeneration primarily occurs secondary to the rupture of intralesional arteries or venous thrombosis, leading to haemorrhagic infarction of leiomyomas. This condition usually manifests with APP, fever, and nausea. Upon US and CT imaging, red degenerated fibroids may appear as large, inhomogeneous, and well-circumscribed intramural masses, with cystic areas and an absence of Doppler signal/flow in infarction areas. MRI examinations may demonstrate a peripheral high-intensity rim on T1-weighted images due to the T1 shortening effects of the methaemoglobin of blood products confined to the thrombosed and dilated vascular structures surrounding the leiomyoma. In gadolinium-enhanced fat-suppressed T1-weighted sequences, red degenerated leiomyomas do not show contrast enhancement (Figure 6) [47,49].

**Figure 7:**
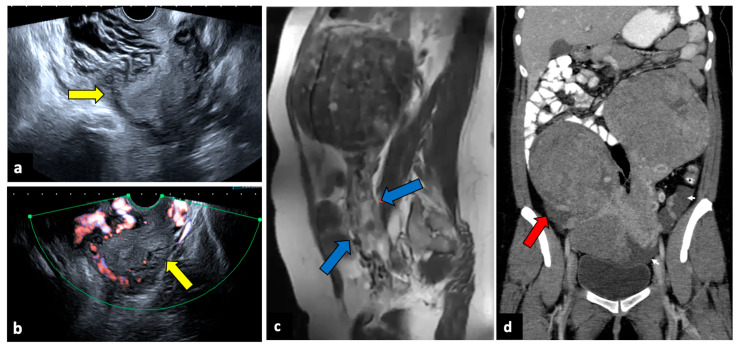
Torsion of a leiomyoma in a 36-year-old woman with acute abdominal and pelvic pain. US (**a**,**b**) shows a normal uterus (yellow arrows) surrounded by multiple dilated vessels. In a previous MR (**c**), a large pedunculated leiomyoma was observed (blue arrows). In a CT scan (**d**), two pedunculated fibroids are seen; the right one is relatively hypodense, with free fluid in the paracolic gutter (red arrow). Surgery findings confirmed the diagnosis of leiomyoma torsion.

The degeneration or torsion of uterine leiomyomas usually occurs in subserosa and pedunculated fibroids, and it is a rare but potentially life-threatening cause of APP. The severity of symptoms depends mainly on the degree of rotation: if pedicle torsion is partial and spontaneously untwisting, symptoms are usually mild; nevertheless, a complete torsion leads to venous stasis, with oedema and congestion, and then causes compression of the arterial blood supply. Obstruction of the arterial network produces haemorrhagic necrosis. If not treated, massive bleeding or peritonitis may occur [50]. US, CT, and MRI are all imaging tools that are useful for fibroids torsion. The most typical imaging findings are a para-uterine mass with a twisted pedicle, vascular flow absence, and mild free abdominal fluid (Figure 7) [50,51].

**Figure 8:**
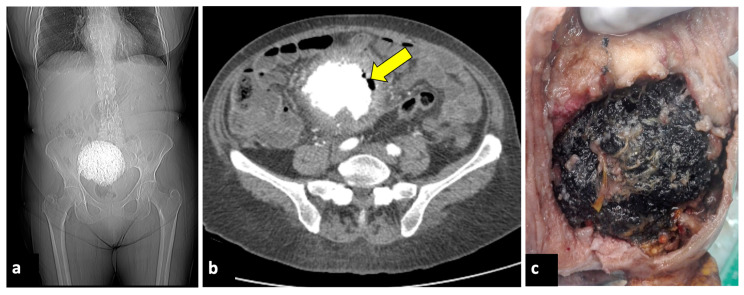
Pyomyoma in an eighty-year-old woman with a history of diabetes and steroid treatments who presented at the emergency department with fever, leucocytosis, and abdominal cramps for a week. A previous X-ray (**a**) shows a calcific uterine myoma. Axial CT scan (**b**) demonstrated the presence of air within the myoma (yellow arrow), eliciting the suspicion of an infected myoma. The patient underwent a hysterectomy (**c**), and the pathological findings confirmed the diagnosis of pyomyoma.

Infection of leiomyomas (pyomyoma) is an infrequent complication of uterine fibroid but is associated with high morbidity and mortality. It may occur in postmenopausal women, during pregnancy, in the postpartum period, or as a complication of uterine artery embolisation for treating leiomyomas [52,53]. Its clinical symptoms usually include fever and PAA. The diagnosis of pyomyoma is tricky because of its insidious presentation and lack of reported imaging and clinically typical findings. However, the presence of air within the myoma at CT evaluation is a crucial finding when there is a suspected infection (Figure 8). MRI may reveal a uterine mass with an inhomogeneous central hyperintensity component, suggesting the presence of blood products, necrotic tissue, and purulent fluids. Air components within the lesion show low signal intensity on T1, T2, and DWI [54].

### 2.5. Endometriosis

Endometriosis, defined as the presence of functional endometrial tissue outside the uterine cavity, is a frequent cause of chronic and cyclic pelvic pain [55,56]. Nevertheless, it can also present with APP. The most common acute complications of endometriosis have an intestinal aetiology, such as obstruction, perforation, and acute appendicitis caused by endometriosis fibrotic implants. Other acute endometriosis manifestations include endometrioma ruptures, endometrioma superinfection, PID with tubo-ovarian abscess, and urinary tract obstruction [57,58,59,60]. MRI is considered the reference standard imaging modality for the diagnosis of endometriosis [61,62]. However, CT is usually the preferred imaging modality when an acute complication of endometriosis occurs in an ED setting.

In the case of gastrointestinal or urinary tract complications in an emergency setting, endometriosis is rarely identified as an aetiologic factor on imaging, even if it should be suspected in the context of known endometriosis with intestinal or urinary tract involvement. The presence of stellar, mildly enhanced, infiltrative, and fibrotic lesions in suggestive areas in female patients of reproductive age may help doctors reach the correct diagnosis [57,58]. During MRI, the presence of a hyperintense area on T1-weighted fat-saturated sequences within the lesion can suggest the diagnosis of endometriosis [44].

The rupture of an endometrioma is an infrequent complication. CT signs are similar to those of a corpus luteal cyst rupture, with spontaneously dense ovarian masses and ascites. MRI usually shows a distorted endometrioma and free peritoneal fluid with a high T1 signal. In the case of endometrioma superinfection, CT usually shows a nonspecific hypodense cystic adnexal with adjacent fat stranding. During the MRI, the superinfection of an endometrioma is suggested by the presence of thickened and enhancing walls of the lesion, an increase in size of an already known endometrioma, the loss of the characteristic high signal in T1 and the shading sign in T2, and a marked restriction of diffusion in DWI [58].

### 2.6. Complicated Ovarian Teratomas

**Figure 9:**
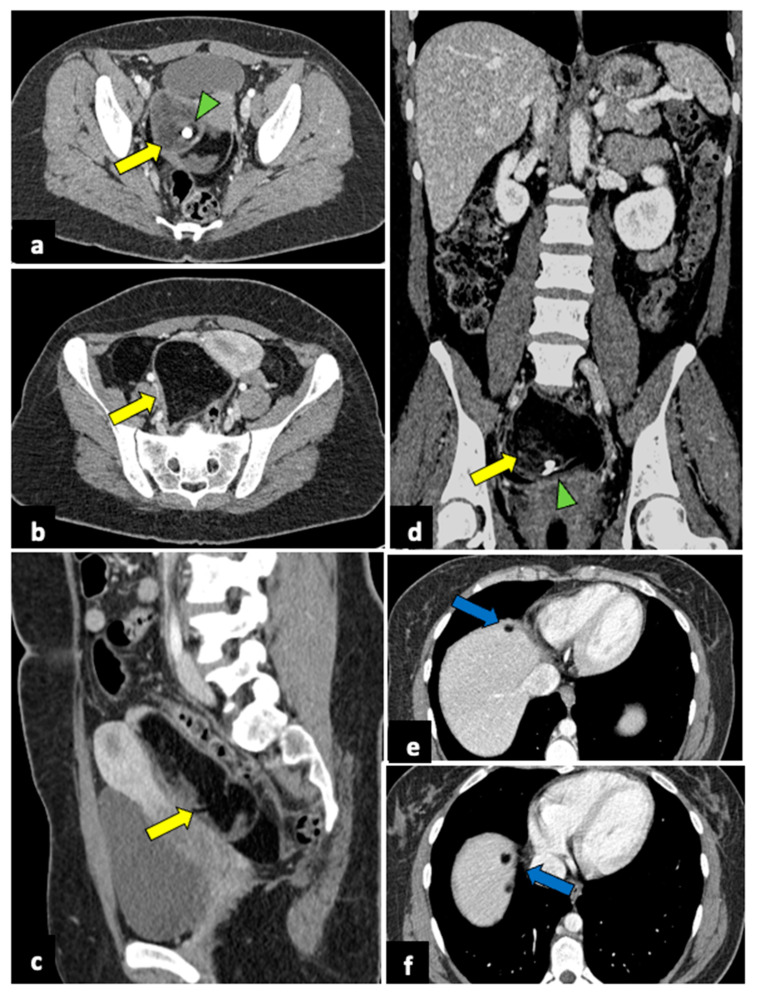
Ruptured ovarian teratoma in a thirty-eight-year-old woman presenting with pelvic pain and fever. CT axial (**a**,**b**), coronal (**c**), and sagittal (**d**) planes reveal a sizeable pelvic mass (yellow arrows) composed of fat and fluid with discontinued walls (red arrow) and an interior tooth-like calcification (green arrowheads). Subphrenic fatty implants (blue arrows) are also observed (**e**,**f**). After adnexectomy, histology confirmed the diagnosis of right mature teratoma.

Mature ovarian teratoma, also known as a dermoid cyst, is the most common benign tumour in premenopausal females [63]. Although it is typically asymptomatic, teratoma may undergo several complications, such as adnexal torsion, rupture, malignant transformation, or infection. The incidence of spontaneous teratoma rupture is thought to range from 0.3% to 2.5% [64]. The discharge of fatty content into the abdominal cavity can cause aseptic peritonitis. Ruptured ovarian teratomas can be suspected when imaging shows discontinuity of the cyst wall, an irregular shape of the teratoma, extra-tumoral fatty nodules, and ascites. CT is the most sensitive technique for detecting intraperitoneal fatty nodules, which are commonly observed around the liver surface (Figure 9) [64,65].

### 2.7. Ovarian Hyperstimulation Syndrome and Hyperreactio Luteinalis

Ovarian hyperstimulation syndrome (OHSS) is a side effect of ovarian stimulation therapy used for in vitro fertilisation. Rarely, it may also occur spontaneously during pregnancy [66]. OHSS is characterised by cystic enlargement of the ovaries and a fluid shift from the intravascular to the third space due to increased capillary permeability and ovarian neo-angiogenesis. It is usually a self-imitating condition, presenting with pelvic pain but, in rare cases, can turn into a life-threatening emergency due to hypovolemic shock [67]. Imaging demonstrates bilateral enlarged ovaries with multiple follicular cysts of varying sizes arranged in a “spoke-wheel” pattern. Peritoneal and pleural effusion may also be present [68]. 

Hyperreactio luteinalis (HS) is a condition that shares many clinical and pathogenetic characteristics with OHSS. Both entities are characterised by bilateral clusters of luteinised ovarian cysts that show a characteristic “spoke-wheel” appearance during an US, accompanied by a physiologic flow on colour Doppler velocimetry [69]. However, OHSS is almost exclusively associated with the induction of ovulation by gonadotropin therapy, while HL occurs during pregnancy, particularly in the second and third trimesters. Patients may be asymptomatic or can present with symptoms such as APP, dyspnoea, or signs of virilisation. Unfrequently, severe ovarian enlargement carries the risk of ovarian rupture, OT, and haemoperitoneum [70].

### 2.8. Gynaecological Malignancies

**Figure 10:**
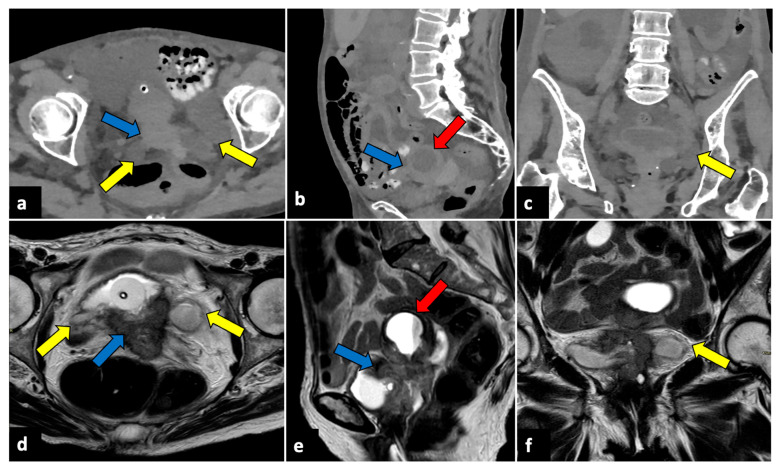
Advanced uterine cervical cancer in a 58-year-old subject attending the emergency department for abdominal pain and haematuria. CT axial (**a**), sagittal (**b**), and coronal (**c**) non-contrast images reveal the presence of a pelvic mass (blue arrows) and ureter causing ureteral obstruction (yellow arrows). The patient further underwent an MRI examination (**d**–**f**), which demonstrated a cervical tumour (blue arrows) invading the parametrium and ureters bilaterally (yellow arrows), the vagina, and the bladder. The uterine cavity was also obstructed and dilatated (red arrows).

Pelvic malignancies, most commonly ovarian, cervical, and endometrial uterine cancers, can induce pain of different intensities and durations. Gynaecological cancer may present with sudden onset in cases of advanced stages, with the invasion of adjacent structures that lead to several complications, such as intestinal perforation, thrombosis, and intestinal or ureteral obstruction (Figure 10) [71,72,73]. 

### 2.9. Ectopic Pregnancy

**Figure 11:**
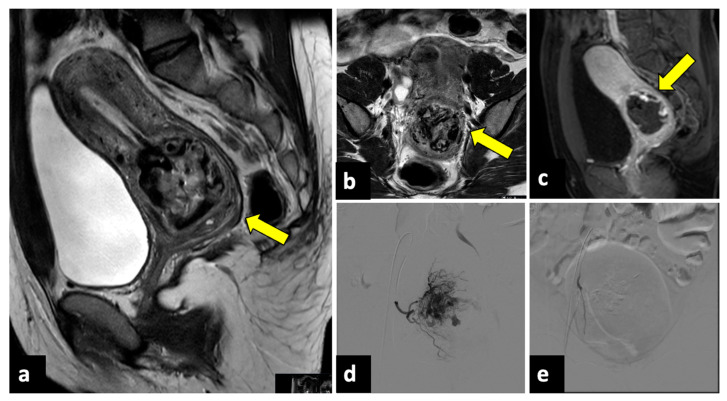
Ruptured cervical ectopic pregnancy in a thirty-three-year-old patient who presented at the emergency department with acute pelvic pain and severe vaginal bleeding. MRI sagittal (**a**) and axial (**b**) T2-weighted, and sagittal T1-weighted early contrast-enhanced fat-saturated (**c**) images show an empty uterine cavity and a ruptured ectopic pregnancy within the cervical canal (yellow arrows). This patient was managed with uterine arteries embolisation. Preprocedural images (**d**) show a hypervascular area in the region of the cervical ectopic pregnancy that is supplied mainly by the right uterine artery. Post-embolisation (**e**) arteriogram demonstrates complete stasis.

Ectopic pregnancy (EP) is the implantation of a fertilised ovum outside the uterine cavity and, due to the risk of massive bleeding, is a potentially life-threatening gynaecological emergency, representing the leading cause of maternal mortality in the first trimester, with an incidence of 5–10% of all pregnancy-related deaths [74,75]. Predisposing risk factors are a previous EP, tubal ligation, a copper intrauterine device (IUD), and artificial insemination. EP most often occurs along the fallopian tubes (97%), especially close to the ampulla. Still, it can only occur in the cervix (Figure 11), in the ovaries, along a previous Caesarean scar, or within the abdominal cavity [76]. EP rupture with massive intra-abdominal bleeding often occurs after 10 to 14 weeks of gestation. A sign of a rupture is severe APP with vaginal bleeding and even haemorrhagic shock. Due to the risk of life-threatening haemorrhages, an early diagnosis of this condition is pivotal. In addition to the pregnancy test, both transabdominal and transvaginal US are essential and show an empty uterine cavity with decidualised endometrium (“decidual sac sign”). The ovarian region should be closely evaluated since a complex extra-adnexal cyst/mass or dilatated tubes are frequently detected in a tubal EP. Usually, a large amount of free peritoneal fluid and/or haemoperitoneum is also observed. The gestational sac appears during the US examination as a ring-shaped lesion with an anechoic centre and a broad, hyperechoic, hypervascular rim (tubal ring sign) [77]. However, in several cases, US cannot definitively exclude EP, and MRI may be helpful by providing a larger field of view and more accurate anatomic localisation. Peri-adnexal masses, haematosalpinx, and haemoperitoneum are the most frequent MRI findings [78,79,80].

### 2.10. Uterine Torsion and Uterine Sacculation

Torsion of a pregnant uterus is an infrequent condition, defined as a rotation of more than 45 degrees around the uterine long axis, most frequently occurring at the level of the uterine isthmus, the transition between the corpus and cervix. Uterine torsion is observed in all age groups during the fertile period and at all stages of pregnancy. Abnormal foetal presentation, large pre-existing leiomyomas, and uterine malformations are considered risk factors [81]. An accurate preoperative diagnosis can be made with both CT and MRI, which demonstrate a whorled structure of the uterine corpus. The uterine twist is usually observed at the isthmus or the cervical and/or upper vaginal region. The uterine cavity is typically filled with fluid, and the uterine vessel appears congested. MR T2-weighted images may also show a twisted pedicle at the level of the uterine isthmus, with the appearance of a whirlpool sign [82]. 

Uterine sacculation is a rare complication of pregnancy that is defined as a transitory sac-like structure or outpouching of the uterus caused by an abnormal forward or backward rotation of the uterine fundus [83]. Without diagnosis and treatment, this condition can lead to uterine rupture, spontaneous miscarriage, intrauterine foetal death, retained placenta, and postpartum haemorrhages. In the emergency setting, US is the most important diagnostic technique, since it can reveal the sac-like structure, with thin and stretched walls compared to the rest of the uterus. MRI can be useful as a second-line imaging method in the case of a nonconclusive US examination [84].

### 2.11. Uterine Rupture

**Figure 12:**
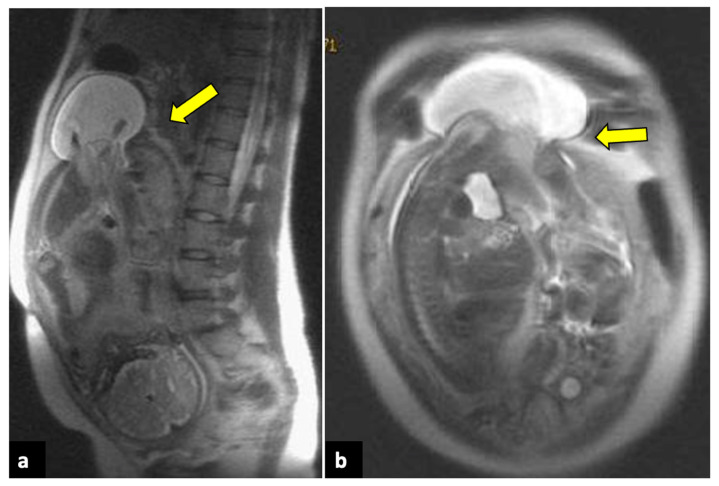
Uterine rupture in a thirty-three-year-old patient presented at the emergency department with acute pelvic pain and abnormal uterine contractions during the 37th week of pregnancy. The patient had a prior history of two previous uterine curettages. MRI T2-weighted sagittal (**a**) and coronal (**b**) images show a complete division of the uterine fundus wall with extrusion of the gestational sac (yellow arrows).

Full-thickness uterine ruptures usually occur during pregnancy, especially in the presence of a previous Caesarean scar. Patients present with APP and haemodynamic instability [85]. US and MRI are both helpful imaging tools for the diagnosis of uterine ruptures. Usually, they depict a protruding portion of the amniotic sac, intraperitoneal foetal parts, extrauterine haematomas, haemoperitoneum, and/or abdominal free fluid (Figure 12) [86].

### 2.12. Placental Disorders

Placental disorders include various entities, such as location, vascular, and implantation abnormalities. US evaluation is the first-line technique for placental evaluation. However, when US findings are equivocal, MRI is an efficient technique to evaluate placental disorders [7,87]. Placental abruption usually manifests with vaginal bleeding and APP, and it leads to three types of haematomas: retroplacental, subchorionic, and subamniotic haematomas. MRI is superior to US in evaluating placenta haemorrhages because of its wider field of view and accurate soft tissue characterisations. T1-, T2- and diffusion-weighted sequences are required to assess intrauterine haematomas and estimate the age of the bleeding [88].

**Figure 13:**
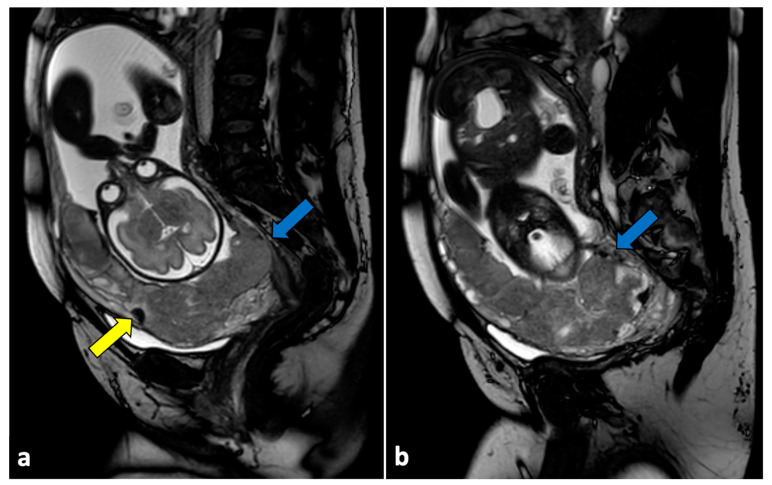
Placenta accreta in a 33-year-old patient at 36 weeks of gestation. Sagittal (**a**,**b**) T2-weighted MR images show a placenta previa (blue arrows) covering the uterine internal os. Placenta has irregular contours and rounded edges, with a single area of intraplacental dark T2 bands (yellow arrow). No interruptions in the thin hypointense myometrial border are seen. Pathologic examination confirmed the diagnosis of placenta accreta.

Moreover, MRI can be useful for assessing abnormal placental villous adherences (placenta accreta, increta, and percreta) [89]. Previous caesarean section and placenta location abnormalities (i.e., placenta previa) are the two major risk factors for placenta accreta. In these conditions, the placenta becomes tethered and does not layer smoothly into uniform thickness. Placental border irregularities, uterine bulging, loss of normal uterine contour, and interruption of the junctional zone are frequent MRI findings of placental implantation disorders [90,91,92,93]. T2-weighted dark intraplacental bands, which are greater and more irregular in thickness than normal septa, are also frequently observed. These bands represent a dense region of fibrous tissue that is consistent with placental infarction [94]. Placenta previa is often associated, resulting from the placental tethering that impedes the normal migration of the placenta during gestation (Figure 13).

## 3. Management of Acute Pelvic Pain 

The prominent role of imaging is to distinguish conservatively treatable APP causes from potentially life-threatening causes (i.e., ectopic pregnancies and cyst ruptures) or emergencies requiring surgery (i.e., ovarian torsion and appendicitis). 

US is the imaging modality of first choice, as it permits driving through the diagnosis to the urinary tract (i.e., lithiasis and pyelonephritis), gastrointestinal (i.e., appendicitis, diverticulitis, obstruction, perforation, and inflammatory diseases), or gynaecological pathologies. Nevertheless, US is an operator-dependent technique and often suffers from technical limitations such as obesity and meteorism. 

When US findings are equivocal, or US cannot be performed due to patients’ discomfort, CT plays a vital role in evaluating female patients with APP. Depending on its availability, MRI is also a valuable diagnostic option. However, in acute situations, it is routinely used mainly in pregnant women or young patients, because it offers excellent spatial and contrast resolution without exposing patients to ionising radiations. For these reasons, in the case of female patients during childbirth age, evaluating serum beta-hCG levels is mandatory [1,2,3,7,95]. 

**Figure 14:**
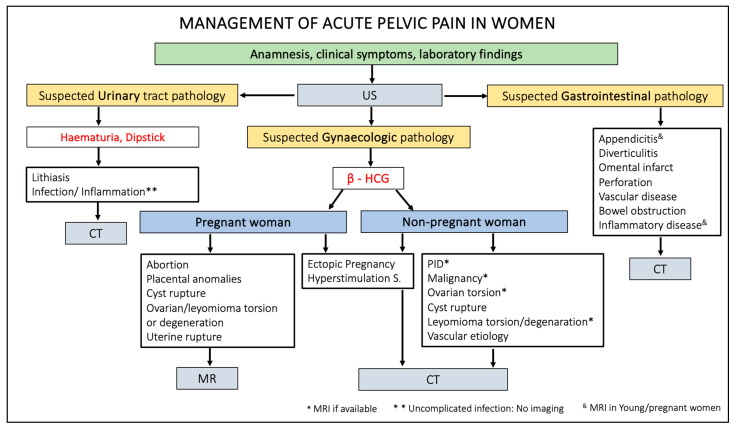
Flowchart of acute pelvic pain management in female patients. US, ultrasound; HCG, human chorionic gonadotropin; CT, computed tomography; MR, magnetic resonance; PID, pelvic inflammatory disease.

Figure 14 summarises the management of patients presenting acute pelvic pain through a practical flowchart. 

## 4. Conclusions

Many gynaecological pathologies may manifest with acute pelvic pain, including pelvic inflammatory disease, uterine and ovarian disorders, endometriosis, and ectopic pregnancies. Diagnostic imaging plays a crucial role in the diagnosing aetiology of APP and distinguishing potentially life- and fertility-threatening conditions from those that can be treated conservatively. Knowledge of the most common imaging findings, obtained through US, CT, and MRI, allows for early and correct diagnosis and assists clinicians in proper treatment planning.

## Figures and Tables

**Table 1:** Gynaecological causes of acute pelvic pain.

Not Pregnant Women	Pregnant Woman
Ovarian torsion Ovarian follicle rupture/haemorrhage Pelvic inflammatory disease (PID) Uterine myomas degeneration/torsion Endometriosis Endometriosis cyst rupture Ovarian hyperstimulation syndrome Gynaecological cancers Ovarian vein thrombosis	Ectopic pregnancy Uterine rupture Uterine torsion Placental disorders Spontaneous abortion

## Data Availability

Not applicable.
